# Non-coding Regulatory Variants in ASD (Autism Spectrum Disorders) Disrupt CTCF Domains and Shape Cell-Type–Specific Neurodevelopmental Landscapes Revealed by Single-Cell Analyses and Cortical Organoids

**DOI:** 10.21203/rs.3.rs-9650619/v1

**Published:** 2026-06-05

**Authors:** Sara Dominguez-Alonso, P Carballo-Pacoret, JR Paul Trotta, J Gonzalez-Peñas, M Parellada, C Arango, A Carracedo, C Rodriguez-Fontenla

**Affiliations:** Universidad de Santiago de Compostela, Santiago de Compostela A Coruña; Universidad de Santiago de Compostela, Santiago de Compostela A Coruña; CNAG-CRG, Barcelona Institute of Science and Technology (BIST); Hospital General Universitario Gregorio Marañón, Instituto de Investigación Sanitaria Gregorio Marañón, Universidad Complutense; Hospital General Universitario Gregorio Marañón, Instituto de Investigación Sanitaria Gregorio Marañón, Universidad Complutense; Hospital Universitario La Paz, Universidad Autónoma de Madrid, CIBERSAM; Universidad de Santiago de Compostela, Santiago de Compostela A Coruña; Universidad de Santiago de Compostela, Santiago de Compostela A Coruña

## Abstract

**Background:**

Autism Spectrum Disorder (ASD) is a complex neurodevelopmental disorder with substantial genetic heterogeneity. While coding variants have been extensively studied, non-coding regulatory variants—which constitute approximately 99% of the human genome—remain largely underexplored. Their functional impact, particularly at the single-cell level and in organoid models, requires further characterization to better understand how these variants contribute to ASD pathogenesis.

**Methods:**

We performed targeted sequencing of 85,394 active cis-regulatory elements (cCREs) from ENCODE v2 in 600 samples (200 trios) previously negative for pathogenic coding variants. Using the Sei deep learning framework, we predicted regulatory impact scores for de novo and ultra-rare inherited variants. Gene mapping of regulatory variants was done using the T Gene tool which assigns target genes by integrating expression data. Differential gene expression was assessed using single-nucleus RNA-seq data from ASD and control brains (n = 64 individuals, ~ 600,000) from Wembley et al, 2023 and temporal gene expression dynamics was characterized in human cortical organoids scRNA seq data across four developmental stages (23 days to 6 months) from the Cortical Organoid Atlas (Broad Institute).

**Results:**

We identified 164 de novo variants in 103 probands and 53,023 ultra-rare inherited variants in 199 probands. Following Sei-based prioritization, 47 de novo variants in 44 probands and 13,258 inherited variants in 198 probands showed significant regulatory impact score > 1.1. De novo variants were enriched in promoter-like sequences (PLS) (p = 0.02), with 53.19% affecting CTCF-mediated chromatin boundaries. Inherited variants showed significant enrichment for CTCF-binding loss (p = 1.55×10^−8^) and demonstrated maternal transmission bias (50.85% vs 49.15%, p = 9.97×10^−5^). Single-cell analysis of the target predicted genes revealed distinct cellular architectures for both gene groups. De novo genes ( POGZ, ROCK2, NFIB ) showed focused dysregulation in oligodendrocytes and specific neuronal populations, while inherited genes ( SYNJ2, UBC, CLU, DLC1 ) exhibited broader effects across multiple cell types. Cortical organoid profiling demonstrated that de novo genes display progressive developmental activation with precise spatiotemporal specificity— POGZ and ROCK2 exhibited bimodal expression peaks at 1 and 6 months in outer radial glia while NFIB showed linear upregulation across progenitor populations. In contrast, inherited genes maintained constitutive expression throughout development, with the exception of CLU, which showed regulated upregulation at later stages. Remarkably, de novo regulatory genes (POGZ, NFIB) are previously identified autism risk genes in large scale sequencing studies, providing validation of dosage sensitivity mechanisms.

**Conclusions:**

Our analysis of regulatory variants in ASD indicates that non-coding mutations significantly contribute to ASD risk by disrupting CTCF domains, leading to alterations in cell type–specific gene regulation. Most de novo regulatory mutations predominantly target genes whose expression is dynamic, varying across different cell types and neurodevelopmental stages, whereas inherited regulatory variants affect constitutively expressed genes across multiple cell types, with stable expression throughout development with the exception of CLU. We also found that genes affected by de novo regulatory variants substantially overlap with those harboring pathogenic coding mutations identified in large-scale sequencing studies. These

## INTRODUCTION

Autism Spectrum Disorder (ASD) is a complex neurodevelopmental disorder (NDD) characterized by challenges in social interaction, communication, and repetitive behaviors. While significant progress has been made in identifying genetic risk factors for ASD, recent research has highlighted the crucial role of non-coding regions of the genome in its etiology. These non-coding elements, which constitute approximately 99% of the human genome, have emerged as important contributors to the genetic landscape of ASD.

Advances in whole-genome sequencing (WGS) technologies and computational methods have enabled researchers to explore the impact of non-coding mutations on ASD risk. Studies have revealed that ASD probands harbor de novo mutations (DNMs) in non-coding regions that disrupt both transcriptional and post-transcriptional regulation, with significantly higher functional impact compared to those found in unaffected siblings. While there is no question that non-coding variation plays a role in ASD, initial WGS studies were limited in scope to less than 100 parent-child trios^1,2^ and it was only 5 years ago that WGS studies significantly expanded, encompassing larger cohorts ranging from 200 to 500 families^3–6^. Given the expectation that non-coding mutations vary widely in functional impact, with only a small fraction likely to exhibit strong effect sizes, detecting associations has proven challenging^7,8^. Under this rationale, power calculations indicate that identifying such signals would necessitate a very large cohort^7,9^. To overcome this, most published studies have restricted to “candidate” non-coding elements, which involves a priori prediction of which regulatory elements of the non-coding genome are important for disease risk^1,10^. In analogy to candidate gene studies, which have consistently struggled to produce robust and replicable results, the selection of candidate regulatory regions is anticipated to yield similar outcomes, due to the multitude of possible combinations involving annotations, cell types, brain regions, and developmental stages^11^. Conversely, other studies have demonstrated that the overall contribution of *de novo* non-coding mutations is comparable to that of loss-of-function (LoF) coding mutations and missense mutations, although not all ASD proband will carry impactful non-coding variation^12^.

More recently, large-scale analyses have begun to clarify the contribution of specific classes of non-coding regulatory regions to ASD. A recent large-scale study examining >16,600 individuals showed that rare inherited variants in evolutionarily informed non-coding regions—including human accelerated regions, validated neural enhancers, and conserved neural enhancer candidates—contribute to ASD risk, particularly in consanguineous families. Functional assays confirmed that patient variants near *IL1RAPL1, OTX1,* and *SIM1* alter enhancer activity, supporting a regulatory mechanism^13^.

However, despite these advances, a major challenge remains: the comprehensive definition of functional non-coding elements and the accurate interpretation of the mutational effects they harbor.

Given these challenges, there is a need for unbiased, tissue-informed strategies that focus on regulatory activity in relevant biological contexts and incorporate robust computational predictors to assess variant function. Such approaches are essential to clarify the contribution of non-coding variation to ASD, identify putative regulatory targets, and define the cellular contexts through which these variants may exert their effects.

Here, we investigate the contribution of de novo and inherited rare non-coding mutations to ASD using an unbiased framework in which cis-regulatory elements (cCREs) were selected solely based on their activity in brain and gastrointestinal tissues, using 926,535 active elements from the ENCODE v2 catalog. We analyzed 600 samples (200 ASD trios previously negative for microarray and WES). Targeted sequencing of these regions enabled the identification of de novo and inherited regulatory variants, which were functionally prioritized using Sei, a deep-learning model of regulatory activity. To infer downstream effects, we mapped variants to putative target genes using T-Gene, integrating ChIP-seq–derived regulatory interactions and tissue-specific expression. Finally, we contextualized these genes using single-cell RNA-seq data from human postmortem brains of individuals with ASD and neurotypical controls, as well as an additional single-cell RNA-seq dataset from cortical organoids spanning multiple developmental stages, to infer their roles across brain development. This integrated strategy aims to systematically characterize rare non-coding variation in ASD and delineate the regulatory mechanisms and cellular contexts through which these variants may contribute to ASD risk.

## MATERIALS AND METHODS

### Cohorts

#### Description

The analysis described herein builds upon the complete sample set examined in Alonso Gonzalez *et al*.^14^, which explored the biological roles of postzygotic and germinal coding mutations in ASD.

DNA extraction from the Spanish ASD samples, consisting of 360 trios, was performed using the GentraPuregene blood kit (Qiagen Inc., Valencia, CA, USA) from peripheral blood.

Participants from Santiago (n = 136) were recruited from the Complexo Hospitalario Universitario de Santiago de Compostela and Galician ASD organizations. Meanwhile, subjects from Madrid (n = 224) were enrolled through the AMITEA program at the Child and Adolescent Department of Psychiatry, Hospital General Universitario Gregorio Marañón.

Inclusion criteria stipulated that only individuals aged 3 years or older were included in the study.

Enrolled participants received a clinical diagnosis of ASD from trained pediatric neurologists or psychiatrists, following the criteria outlined in both the DSM Fourth Edition Text Revision (DSM-IV-TR) and Fifth Edition (DSM-5). Additionally, when deemed necessary, the Autism Diagnostic Observation Schedule (ADOS) and the Autism Diagnostic Interview-Revised (ADI-R) were administered.

All participants (probands, parents or legal representatives) gave their written consent and the study was conducted under the Declaration of Helsinki. The Galician Committee of Research Ethics (Xunta de Galicia) has approved this study under registration number CEIC 2020/400.

Ninety trios from the Spanish cohort were already analyzed by Lim *et al*.^15^. The entire Spanish cohort was included in Satterstrom *et al*.^16^ as part of the Autism Sequencing Consortium (ASC), a large-scale international genomic consortium integrating ASD cohorts and sequencing data from over one hundred investigators. All data generated as part of the ASC were transferred to dbGaP with Study Accession: phs000298.v4.p3.

#### Sample selection

Out of the 360 trios with complete phenotype information, microarray and exome sequencing data, we selected cases with negative results for copy number variants (CNVs) and WES (mutations classified as benign, likely benign or VOUS (variant of uncertain significance)). By selecting cases on the basis of the absence of *de novo* LoF mutations or large *de novo* CNVs in prior WES and microarray data, we are intentionally enhancing the sample for undiscovered non-coding risk variants. After this exclusion, individuals with a syndromic form of ASD were also discarded, in order to avoid ascertainment biases. Following this, samples were randomly selected, leading to a total sample size of 200 trios (100 from Santiago, 100 from Madrid), including data for 39 female probands and 161 male probands. Among the most prevalent comorbidities in our cohort, the following stand out: 41.5% of our patients exhibit ID, 7% have ADHD, and 6.5% experience epilepsy.

#### Selection of the regulatory regions of interest

For the selection of the regulatory regions of interest, we employed the candidate cis-regulatory elements (cCREs) Registry from ENCODE^17,18^ (version 2, https://screen-v2.wenglab.org/). Brain and GI tissues with DNAse-Seq data available were selected, both from adult (n = 14) and embryonic tissue (n = 59). For each tissue, cCREs labeled as “Low-Dnase” were excluded, as they are inactive in the given tissue^17^.

From the total of 926,535 human cCREs, we selected those that exhibit activity in a greater number of the interrogated tissues. Thus, we selected cCREs that were active in 36 or more tissues (n = 85,394 cCREs). (See Additional Supplementary Material)

#### Targeted sequencing

Selected regions were sequenced at the National Center for Genomic Analysis (CNAG) using the KAPA HyperChoice Target Enrichment custom probes.

Samples with sex discrepancies when compared to reported pedigrees were dropped and replaced, along with all other samples from the same trio. Moreover, samples which failed CNAG’s quality control (DNA <100 ng / critical degradation (genomic quality number (GQN) < 3.3) were also removed and consequently substituted, leaving 71 trios from Santiago and 129 from Madrid.

Sequencing reads were aligned to GRCh38/hg38 using the Burrows-Wheeler Aligner^19^. Single-nucleotide variants (SNVs) and small insertions-deletions (indels) (< 50 bp) were discovered using the Genome Analysis Toolkit (GATK)^20^ HaplotypeCaller package version 3.4 (https://github.com/broadinstitute/gatk).

Targeted sequencing data can be found at EGA, Title of the dataset: Targeted sequencing data of regulatory regions in 200 Spanish ASD trios. https://ega-archive.org/studies/EGAS50000001395

#### Data processing

Raw results were downloaded as single-sample gVCFs (genomic variant call format). All individual gVCFs were combined into one multisample gVCF using GATK version 4.2.2.0.Then, SNVs and indels were jointly called across all samples producing a final multi-sample VCF file (format VCFv4.2). Variant call accuracy was estimated using the GATK Variant Quality Score Recalibration (VQSR) method, following the recommendations provided in the GATK4 Best Practice Workflow for SNP and Indel calling^21^.

#### Identification of regulatory variants

A summary of the quality control and data cleaning procedure is depicted in Suppl Figure 1. For variant and genotype quality control (QC), as well as optimization of *de novo* variant discovery, we utilized the filtering process established by Satterstrom *et al*.^16^. This process was previously optimized and implemented in an ASD cohort, and we adapted it for our dataset. For inherited variant discovery, we implemented the pipeline defined in Wilfert *et al*.^22^ Detailed Hail functions and genotype quality control as well as details for de novo and inherited variants can be found in the Supplementary Material Methods section.

#### Regulatory impact prediction

*De novo* and inherited variants were evaluated to determine their impact on regulatory activities using the Sei framework^18^ (https://github.com/FunctionLab/sei-framework).

Sei provides a comprehensive mapping of any given sequence to regulatory activities, classified in 40 distinct sequence classes. It further provides quantitative scores that represent changes in regulatory activities^18^. Within this context, the Sei framework was utilized to acquire sequence class score predictions for the final call set of *de novo*/inherited variants.

For every mutation, we predicted the sequence class scores for both the reference and alternate alleles and computed the sequence class-level variant effect as the predicted scores for the alternate allele subtracting the scores for the reference allele.

For mutations with a strong effect in a different sequence class than the originally assigned sequence class (absolute value higher than the original sequence class by > 1.1 difference) we reassigned the mutation to the sequence class with the strongest effect.

Moreover, variants were annotated in terms of positive effect predictions (increase in the assigned activity of a sequence) or negative effect predictions (decrease in the activity).

These filtering yielded 47 *de novo* variants, in 44 ASD probands with changes in sequence class activity and 13,258 ultra-rare inherited variants in 198 probands (Supplementary Tables 1, 2, 3).

#### Candidate gene elucidation by physical distance and expression data

To identify target genes based on genomic physical distance, we used the -closest function from the bedtools suite (https://bedtools.readthedocs.io/en/latest/content/tools/closest.html)^23^.

To identify target genes for TRs within cCREs, we used the T-Gene tool (https://memesuite.org/meme/doc/tgene.html), which infers regulatory relationships by combining genomic closeness and ENCODE ChIP-seq data (hg19). We customized T-Gene output to include all potential regulatory links by selecting the “Link” option, which avoids discarding cCREs that may share target genes^24^.

As input for both T-Gene and *bedtools*, we used the set of cCREs harboring 47 de novo mutations, including all cCREs prioritized by Sei based on their predicted regulatory impact (see Suppl Table 1, Suppl Table 4).

For inherited mutations, due to the large number of variants per individual and the generally lower predicted effect of each one, we applied a prioritization strategy using the top 5% of variants based on their Sei scores (662 mutations in 648 unique cCREs) (95th percentile threshold; see Supplementary Table 2.1, 2.2). We customized T-Gene output to include all potential regulatory links by selecting the “Link” option, which avoids discarding cCREs that may share target genes (see Suppl Table 2, Suppl Table 5).

Subsequently, gene lists were filtered considering Correlation and Distance p-value (CnD p-value) < 0.05 of T-Gene. For distance vs expression gene comparison, we filtered T-gene output taking in account only the gene linked the most (lowest CnD p-value) for each cCRE for both cohorts (Suppl Table 6, Suppl Table 7).

To carry out the differential single cell analysis, de novo from T-Gene were filtered to retain only those links with a combined distance and correlation and Distance p-value (CnD p-value) < 0.05. In the case of inherited genes results from T-Gene were filtered to retain only those links with a combined distance and correlation and Distance p-value (CnD p-value) < 0.05 and q-value > 0.1 (Suppl Table 8, Suppl Table 9).

#### Single-cell gene differential gene expression analysis ASD vs controls

In order to carry out a single cell differential gene expression analysis in neuronal and glial cells, we used ASD scGENE Portal (http://solo.bmap.ucla.edu/asdscgene/), resource that uses single nucleus RNA-seq data from 33 individuals with ASD and 31 control subjects, comprising nearly 600,000 cell nuclei. This dataset, published by Wamsley et al., 2023, enables transcriptomic comparisons across a wide range of brain cell types^25^. We used this resource to explore whether any of the 39 associated de novo genes and 74 inherited genes as described above, were expressed in specific neuronal or glial subtypes, and whether their expression was altered in ASD compared to controls. To this end, we examined cell-type-specific expression patterns and differential expression across major cell populations. There were 35 cell types identified by single-nucleus RNA sequencing (snRNA-seq), divided in 8 major cell types: oligodendrocyte progenitor cells (OPCs); astrocyte (ASTRO); microglia (MG); endothelial cells (ENDO); inhibitory neurons (INT); excitatory neurons (EXT); oligodendrocyte (ODC) and blood-brain barrier (BBB).

Using de novo and inherited genes identified by T-gene with a CnD p-value < 0.05, we looked for genes showing significant differential expression between ASD cases and controls defined by an FDR < 0.05, and an absolute log fold change (|logFC|) > 0.3 (Suppl Table 10, Suppl Table 11).

#### Analysis of Gene Expression in Cortical Organoids Across Developmental Stage

Genes previously identified as differentially expressed between Autism Spectrum Disorder (ASD) cases and neurotypical controls in our transcriptomic analyses were selected for downstream characterization in human cortical organoid datasets. Publicly available single-cell RNA-sequencing (scRNA-seq) data from the *Cortical Organoids Atlas* (Broad Institute Single Cell Portal, https://singlecell.broadinstitute.org/single_cell/study/SCP1756/cortical-organoids-atlas) were used to examine the temporal and cell-type–specific expression patterns of these genes across organoid development^26.^ We have employed data from 4 different developmental time points: 23 days, 1 month, 3 months, and 6 months in vitro. These stages approximate key periods to early neurogenesis, mid-fetal corticogenesis, and the emergence of more mature neuronal populations^27^.At 23 days, organoids mainly contain early neural progenitors, including aRG (apical radial glia), intermediate progenitors, cortical hem populations, preplate/subplate cells, subcortical neurons and their progenitors, as well as lineages derived from the neural crest and neural placode. At 1 month, organoids show a transition toward more defined neuronal populations, including aRG, Cajal–Retzius cells, intermediate progenitors, subcortical neurons, and the first newborn excitatory neurons. At 3 months, major cortical neuronal types emerge, particularly CPN (callosal projection neurons) and CFuPN (corticofugal projection neurons), along with oRG (outer radial glia), a basal radial glia type that plays a key role in human cortical expansion and late cortical neurogenesis. By 6 months, organoids display increased maturation, with prominent populations of astroglia, glial precursors, oRG and oRG-like subpopulations, progenitors, immature inhibitory neurons, and more differentiated excitatory neurons.Processed count matrices, metadata, and cell-type annotations were accessed through the Broad Institute Single Cell Portal interface. We utilized the preprocessed and annotated version of the dataset provided by the authors, which includes quality-controlled cells, normalized expression matrices, and curated cell-type labels. At each developmental stage, we used the cell-type annotations provided in the dataset.

## RESULTS

### Dataset

We analyzed targeted sequencing data in a cohort of 200 ASD trios, with a ratio of ~4:1 male probands to females (161 males, 39 females; male-to-female ratio = 4.13), in line with previous estimates^28^.

Based on the evidence of GI and brain tissues playing a role in ASD’s etiology^29^, regions were selected according to their activity in these tissues. This yielded a total of 85,394 cCRES active in 25 brain-tissue samples and 48 GI-tissue samples, spanning a total of 21.35 Mb of the genome (*i.e.,* 0,68% from the total length, 0,70% from the non-coding length)^30^.

**Figure 1:**
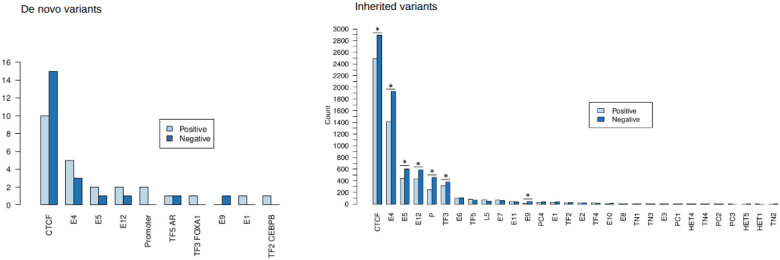
Pie chart depicting the classification of cCREs. Panel A illustrates the distribution of cCREs in the ENCODE registry, while Panel B displays the percentages within our dataset's selection.

The vast majority of selected cCREs corresponded to the ENCODE classification of distal/proximal enhancer-like signature (n = 61,822; 72.40%) whereas promoters were represented to a lesser extent (n = 20,306; 23.78%) (Figure 1), as expected in the basis of the one-to many relationship between promoters and enhancers.

### *De novo* variants

**Figure 2:**
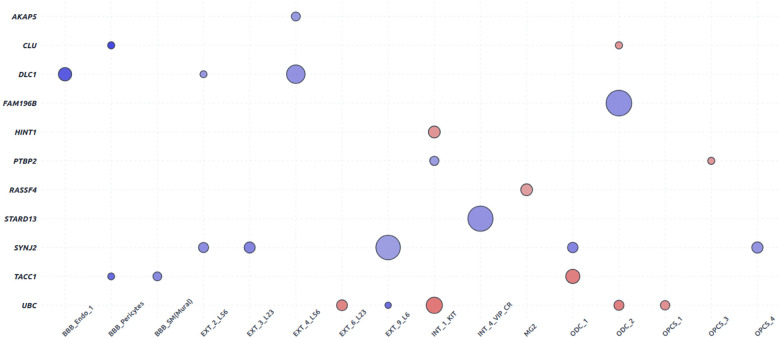
Pie chart representation of *de novo* and inherited variants based on cCREs’ classification. Panel A illustrates the classification for *de novo* variants, while Panel B depicts the classification for inherited variants.

From the trio-based data, we identified 164 rare *de novo* variants in the selected candidate regulatory regions (allele frequency ≤ 0.1% in our dataset and in the non-psychiatric of gnomAD), with 51,50% of cases carrying at least one such variant (103 ASD probands; $\bar{\text{x}}$ = 1.59 ± 0.84 variants/child). These variants were present inside an ELS cCRE (both distal and proximal) in 67.08% of the instances, whereas 31.10% were harbored in PLS. The remaining variants were located inside a CTCF-only cCRE (1.22%) and DNAse-H3K4me3 cCREs (0.61%) (Figure 2A).

### Inherited variants

**Figure 3:**
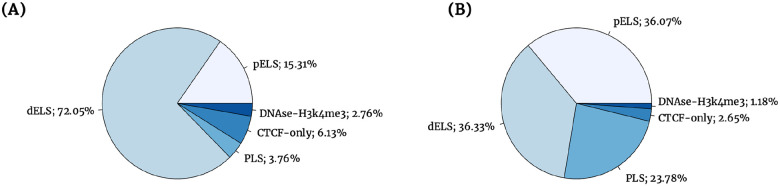
Counts of de novo and inherited variants across regulatory classes. Variants are grouped by regulatory class and categorized according to positive or negative regulatory effect scores. *Significant differences between the number of variants with positive versus negative scores were assessed using a two-sided binomial test*.

To study ultra-rare inheritance, we included variants with an allele frequency ≤ 2.5×10^−3^ in the parent population and selected those transmitted to the child. After removing one outlier family with inconsistencies in respect to the average number of mutations per family, 53,023 inherited variants were detected in 199 cases ($\bar{\text{x}}$ = 266.45 ± 68.93 variants/child). These variants were present inside an ELS cCRE in 67.02% of the instances, whereas 29.45% were harbored in PLS cCREs. The remaining variants were located inside a CTCF-only cCRE (2.39%) and DNAse-H3K4me3 cCREs (1.14%) (Figure 3B).

Of note, we observed 26,960 informative sites with a mother-proband inheritance, and 26,063 informative sites with a father-proband inheritance (50.85% *versus* 49.15% of the instances, respectively). The observed differences between these two inheritance patterns were statistically significant (p = 9.97 × 10^−5^; two-sided binomial exact test), consistent with the maternal transmission bias observed for large and small CNVs and inherited private truncating SNVs^1,31–33^. This bias remained significant even when restricting to autosomal chromosomes (p = 7.8 × 10^−3^, two-sided binomial exact test).

Additionally, when restricted to the X chromosome, most of the variants were maternally inherited (97.98% with maternal inheritance pattern *versus* 2.02% with paternal inheritance pattern), as would be expected. Intriguingly, all variants exhibiting an X-linked maternal inheritance pattern were consistently transmitted to an affected son (p = 2.2 × 10^−16^; two-sided binomial exact test) and in any case, to a daughter.

### Variant enrichment in cCREs (candidate cis-Regulatory Elements)

To assess potential statistical enrichments or depletions in the number of *de novo* and inherited variants per cCRE category, we conducted two-sample proportions tests.

Comparative analysis with the proportion of each cCRE in the original selection (Figure 2 B) revealed an enrichment of *de novo* and inherited variants in PLS cCREs (p-value for proportion test = 0.02 and < 2.2 × 10^−16^, respectively) (Table 1). Additionally, inherited variants were depleted in CTCF cCREs (p-value = 1.46 × 10^−3^ for the proportion test) and enriched in pELS cCREs (p-value = < 2.2 × 10^−16^ for the proportion test).

### Effect of genetic variants in gene regulation

In order to prioritize variants for their potential impact on gene regulatory activities, we used Sei, a new deep learning sequence model that enables the interpretation of genetic variants.

#### *De novo* variants

*De novo* variant class scores ranged from 0.07 to 6.68, with 28.66% (n = 47) of the variants yielding absolute differences higher than the stipulated class score > 1.1 difference to consider a variant of high regulatory impact (Supplementary Table 1). These variants were assigned to 10 different regulatory classes in Sei.

Of note, the 47 variants were present in 44 probands, with 3 of them harboring 2 different variants in separate chromosomes ($\bar{\text{x}}$ = 1.07 ± 0.25 *de novo* variants/child).

Half of these variants (53.19%) represented the class CTCF, which demarcates topological loop boundaries^34^. Out of the 25 variants assigned to the regulatory class “CTCF CTCF-Cohesin”, 24 were classified in ENCODE as PLS/dELS/pELS-CTCF bound, and one variant was categorized as CTCF-only.

For the other regulatory classes, variants were inside ENCODE cCREs defined as PLS/dELS/pELS (CTCF bound or not) without following any apparent trend (*i.e*., no regulatory class was significantly correlated with a specific type of cCRE) (data not shown).

#### Inherited variants

Inherited variant class scores ranged from 0.006 to 41.41, with 25.00% (n = 13,258) of the variants yielding absolute differences higher than > 1.1 (Supplementary Table 2) in 30 different regulatory classes.

The average number of inherited variants was $\bar{\text{x}}$ = 66.96 ± 17.41, and the maximum observed number of inherited variants in a proband was 182.

The CTCF class was the most widely represented in our dataset (40.62% of the variants). In line with the results from *de novo* variants, only 162 variants were present in a CTCF-only cCRE, while the rest were classified in ENCODE as PLS/dELS/pELS/DNase-H3K4me3 CTCF bound or not. Moreover, there were no specific correlations between ENCODE’s cCRE classification restricted to any Sei-assigned class groups, except for CTCF-only cCREs (p-value = 1.79 × 10^−6^ for the proportion test) (Supplementary Table 3).

#### Differences in gene regulation for inherited and *de novo* variants

*De novo* variants did not yield any significant differences in terms of positive / negative scores neither for the CTCF category (10 variants gained affinity for CTCF binding, and 15 lost this affinity; p-value = 0.42, two-sided binomial test), nor for the rest (Table 2, Figure 3).

For inherited variants, however, we found a significant enrichment in negative scores within the CTCF regulatory class (*i.e*., a significant decrease in the affinity for CTCF; p-value = 1.55 × 10^−8^, one-sided binomial test) (Table 2, Figure 3). Besides the CTCF class, 6 additional regulatory classes yielded a significant enrichment in negative scores (Table 2).

### Candidate gene elucidation by physical distance and expression data

When comparing target genes assigned to cCREs carrying de novo mutations based on physical proximity versus those assigned using gene expression and ChIP-seq data through the T-Gene, we found that 7 out of 39 genes were not shared between the two lists. This indicates that approximately 17.9 % of the genes assigned based on expression data differed from those assigned by genomic distance alone.

When we carried out the same analysis using the cCRES carrying the top 5% inherited mutations prioritized by Sei score, we found that 420 out of 648 genes were shared between those assigned by genomic and those assigned by expression data. This indicates that 64.8% of the genes overlapped between both lists, while 35.2% differed.

The gene list obtained through expression-based assignment using T-Gene allowed us to further prioritize candidate genes detailed in the Materials and Methods section. This prioritization yielded a total of 39 de novo candidate genes and 74 inherited candidate genes that were employed in subsequent single cell analysis (Suppl Table 8, Suppl Table 9).

### Gene differential gene expression analysis using snRNAseq from ASD brain vs controls De novo genes

**Figure 4:**
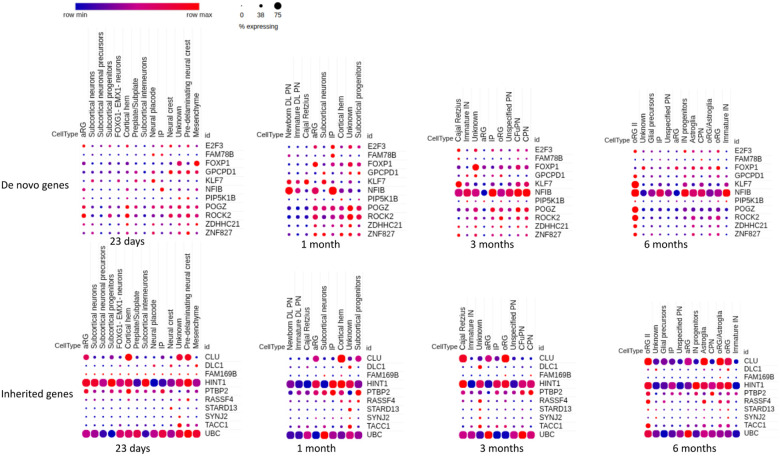
Dot plot displaying cell type-specific differential de novo-gene expression in ASD brain tissue vs controls using RNA-seq cell data.

Differential gene expression analysis using snRNA-seq data and the set of prioritized genes obtained from the previously described analysis revealed a subset of genes that were differentially expressed between ASD and control brains. *POGZ* demonstrated the most robust statistical significance (FDR = 2.90×10^−8^) and largest effect size (logFC = −0.638) among downregulated genes, occurring specifically in oligodendrocytes. *ROCK2* **e**xhibited cell type-dependent regulation, with significant upregulation in BBB-associated cells contrasting with downregulation in excitatory neurons, suggesting context-specific functional roles. *NFIB* showed consistent downregulation across multiple cell types, indicating potential widespread disruption of this transcriptional program (Suppl Table 10, Figure 4).

Dot plot displaying differential gene expression patterns across brain cell types comparing ASD and control samples. Dot size is proportional to the statistical significance, calculated as -log_10_(p-value), with larger dots indicating more significant findings. Dot color represents the direction and magnitude of expression change: red indicates upregulation in ASD (positive log fold change), blue indicates downregulation in ASD (negative log fold change), and color intensity reflects the absolute magnitude of the log fold change. Gene names (y-axis) are displayed in italics. Cell type abbreviations (x-axis): BBB_Endo_1, blood-brain barrier endothelial cells type 1; BBB_MG3, blood-brain barrier microglia type 3; BBB_Pericytes, blood-brain barrier pericytes; EXT_1_L4, excitatory neurons layer 4; EXT_5_L56, excitatory neurons layer 5/6; EXT_6_L23, excitatory neurons layer 2/3; EXT_9_L6, excitatory neurons layer 6; INT_4_VIP_CR, interneurons VIP-positive/calretinin-positive; INT_5_SST, interneurons somatostatin-positive; ODC_1, oligodendrocytes type 1; OPCS_3, oligodendrocyte precursor cells type 3. Dashed grid lines indicate the position of each gene-cell type intersection. n = 15 significant gene-cell type associations across 11 unique genes and 11 cell types. Gene names are shown in italics on the y-axis, and cell type abbreviations are displayed vertically on the x-axis. Cell types are described in the Methods section. Only gene-cell type pairs with FDR ≤ 0.3 are displayed.

### Inherited genes

*SYNJ2* emerged as the most consistently dysregulated gene, showing downregulation across five cell types, with the strongest effect in layer 6 excitatory neurons (logFC = −0.306, FDR = 1.88×10^−9^). *UBC* showed bidirectional, cell type-specific regulation: it was downregulated in layer 6 excitatory neurons and upregulated in KIT^+^ interneurons, oligodendrocytes, layer 2/3 excitatory neurons, and oligodendrocyte precursors. Multiple BBB-associated genes showed robust alterations. *CLU* displayed the largest effect size, strongly downregulated in pericytes (logFC = −0.699). *DLC1* was markedly reduced in endothelial cells (logFC = −0.606), and *TACC1* was consistently downregulated in pericytes and mural cells. Layer-specific excitatory neuron changes included *DLC1* downregulation in two layer 5/6 populations and *AKAP5* downregulation specifically in EXT_4_L56. VIP^+^/CR^+^ interneurons showed highly significant *STARD13* downregulation.

Oligodendrocyte lineages displayed both up and downregulation: *CLU* was upregulated in mature oligodendrocytes (ODC_2), *TACC1* was upregulated in ODC_1, and *FAM196B* showed strong downregulation in *ODC_2*. Microglia exhibited selective activation, with *RASSF4* upregulated in MG2.

**Figure 5:**
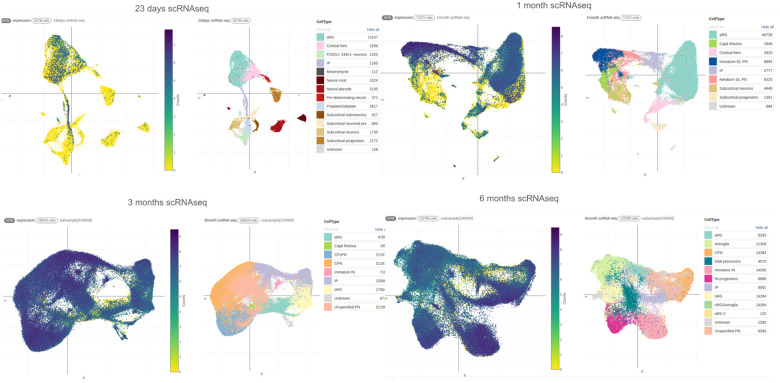
Dot plot displaying cell type-specific differential of inherited-gene expression in ASD brain tissue vs controls using RNA-seq cell data.

Functionally, the most altered genes implicate diverse biological processes: *SYNJ2* (phosphoinositide metabolism and synaptic vesicle recycling), *UBC* (proteostasis), and the *Rho GTPase regulators DLC1* and *STARD13* (cytoskeletal organization). These findings demonstrate complex cell type-specific transcriptional networks in ASD brain tissue, characterized by both consistent directional changes across multiple cell types and context-dependent bidirectional regulation of individual genes within distinct cellular populations (Suppl Table 11, Figure 5).

Dot plot displaying differential gene expression patterns across brain cell types comparing ASD and control samples. Dot size is proportional to the statistical significance, calculated as -log_10_(p-value), with larger dots indicating more significant findings. Dot color represents the direction and magnitude of expression change: red indicates upregulation in ASD (positive log fold change), blue indicates downregulation in ASD (negative log fold change), and color intensity reflects the absolute magnitude of the log fold change. Gene names (y-axis) are displayed in italics. Cell type abbreviations (x-axis): BBB_Endo_1, blood-brain barrier endothelial cells type 1; BBB_MG3, blood-brain barrier microglia type 3; BBB_Pericytes, blood-brain barrier pericytes; EXT_1_L4, excitatory neurons layer 4; EXT_5_L56, excitatory neurons layer 5/6; EXT_6_L23, excitatory neurons layer 2/3; EXT_9_L6, excitatory neurons layer 6; INT_4_VIP_CR, interneurons VIP-positive/calretinin-positive; INT_5_SST, interneurons somatostatin-positive; ODC_1, oligodendrocytes type 1; OPCS_3, oligodendrocyte precursor cells type 3. Dashed grid lines indicate the position of each gene-cell type intersection. n = 15 significant gene-cell type associations across 11 unique genes and 11 cell types. Gene names are shown in italics on the y-axis, and cell type abbreviations are displayed vertically on the x-axis. Cell types are described in the Methods section. Only gene-cell type pairs with FDR ≤ 0.3 are displayed.

### Analysis of Gene Expression in Cortical Organoids Across Neurodevelopmental Stage

#### De novo gene patterns

**Figure 6:**
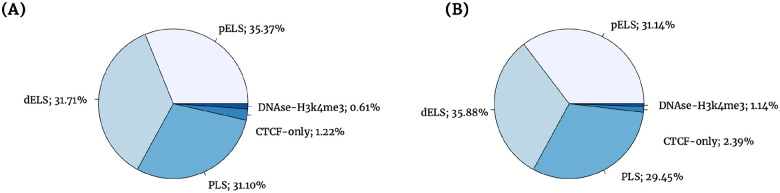
Temporal Expression Patterns of De Novo and Inherited Genes in Developing Cortical Organoids. Single-cell RNA sequencing expression dot plots across four developmental timepoints (23 days, 1 month, 3 months, 6 months). Top row shows de novo genes; bottom row shows inherited genes. Dot size represents the percentage of cells expressing each gene. Dot color indicates average expression level (blue = low, red = high). De novo genes show progressive activation and cell-type specialization over time. Inherited genes maintain constitutive expression throughout development, except *CLU* which shows regulated upregulation at later timepoints (3–6 months).

These genes show a clear temporal progression. At 23 days, expression is sparse and scattered across cell types. By 1 month, we can see increased expression in specific cell types. This intensifies further at 3 months, and by 6 months, many of these genes show robust expression in particular cell populations. This indicates activation and specialization over developmental time. Specifically*, NFIB* shows a striking progressive increase in expression specifically in certain cell types across the timepoints. *POGZ* and *ROCK2* demonstrated a distinctive bimodal expression pattern specifically in ORG II cells. Both genes showed elevated expression at 1 month of development, followed by a relative decrease at 3 months, and then re-emergence of high expression by 6 months. This biphasic pattern suggests *POGZ* and *ROCK2* are engaged during two distinct developmental phases of ORG II cell specification and maturation. The initial upregulation at 1 month may reflect early ORG II identity specification, while the re-engagement at 6 months likely corresponds to functional maturation and specialization of ORG II populations (Fig 6).

#### Inherited gene patterns

In contrast to de novo genes, inherited genes (*CLU, DLJ1, FAM168B, HINT1, PTBP2, RPLP0, STARD13, SYN2, UBC*) displayed fundamentally different expression dynamics. Most inherited genes (*UBC, STARD13, SYN2, RPLP0, HINT1, PTBP2, FAM168B, DLJ1*) remained consistently and robustly underexpressed or overexpressed across all cell types and throughout all developmental timepoints from 23 days through 6 months.

*CLU* stood out as an exception within the inherited genes panel. Unlike the other inherited genes that maintained strong, consistent expression, *CLU* showed notably reduced expression relative to its peer group. This expression pattern suggests *CLU* has a more specialized or regulated role compared to the constitutively active inherited genes. However, *CLU* expression appeared to increase at later timepoints (3–6 months) relative to earlier stages, indicating potential upregulation during later stages of organoid maturation despite maintaining lower baseline expression than other inherited genes (Fig 6).

De novo genes represent genes progressively activated during organoid development with increasing cell-type specialization, particularly *NFIB* which shows linear progressive upregulation and *POGZ/ROCK2* which show specialized bimodal dynamics in ORG II populations. In contrast, inherited genes represent constitutive developmental machinery with the exception of *CLU*. This is why we aim to examine the UMAP projection for these genes in greater detail.

**Figure 7:**
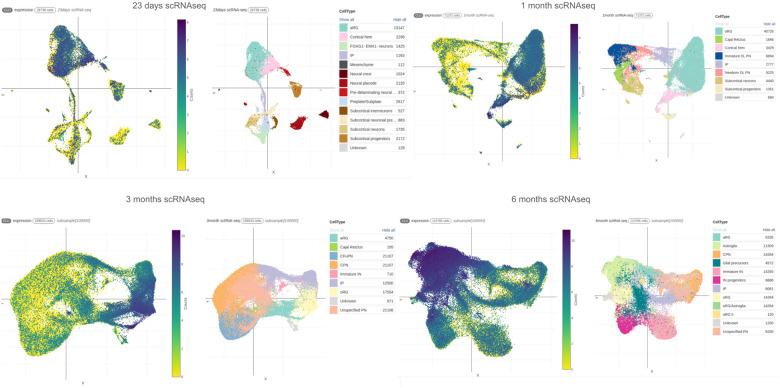
NFIB UMAP projections of single-cell transcriptomes across four developmental timepoints (23 days, 1 month, 3 months, and 6 months postnatal). For each timepoint, the left panel shows the distribution of cells colored by gene expression density (purple blue = high, yellow = low), while the right panel displays cells colored by assigned cell type identity. Each point represents an individual cell, with spatial proximity indicating transcriptional similarity. The progressive expansion and refinement of cell type clusters across developmental stages demonstrates dynamic cellular maturation and increasing cellular heterogeneity. N = cells per timepoint. scRNAseq, single-cell RNA sequencing; UMAP, Uniform Manifold Approximation and Projection.

At 23 days of development, *NFIB* expression was restricted to discrete cellular populations. UMAP analysis identified NFIB-expressing cells distributed across multiple clusters, with the most prominent signal concentrated in a subset of cells, aRG, IP forming distinct islands of high expression. By one month of development, *NFIB* expression expanded substantially, demonstrating broader cellular distribution across the tissue. The single-cell landscape showed increased representation of NFIB+ cells with more uniform spatial organization compared to the 23-day timepoint. At three months, NFIB expression achieved a more confluent distribution pattern, with large contiguous regions now showing elevated expression levels. This stage appeared to represent a transition toward the mature expression pattern, with expression expanding into previously sparse regions while *maintaining* regional specificity. By six months and through the mature one-month stage, *NFIB* expression demonstrated the most extensive and organized spatial distribution (Fig 7).

Overlay of *POGZ* expression with cell type annotations revealed preferential expression in subsets of cortical cell types. High POGZ expression was observed in clusters corresponding to neural progenitor cells and specific neuronal subtypes, while other populations, including certain mature neuronal classes and non-neuronal cells, showed minimal or absent POGZ expression. The spatial segregation of POGZ-high and POGZ-low populations within the UMAP space indicates that *POGZ* expression correlates with specific transcriptional states and developmental trajectories. This restricted expression pattern is consistent with POGZ's known role as a chromatin regulator involved in lineage specification and neuronal differentiation (Suppl Fig 2). It should be noted that both *POGZ* and *ROCK2* show parallel expression trajectories across developmental time and cell types, also exhibiting high expression at 6 months in the ORG II populations (Suppl Fig 3).

Interestingly, among the inherited genes, *SYNJ2* shows consistently low expression compared to other genes. This suggests that it may be differentially expressed between ASD cases and controls, but does not exhibit significant changes during cortical neurodevelopment.

**Figure 8:**
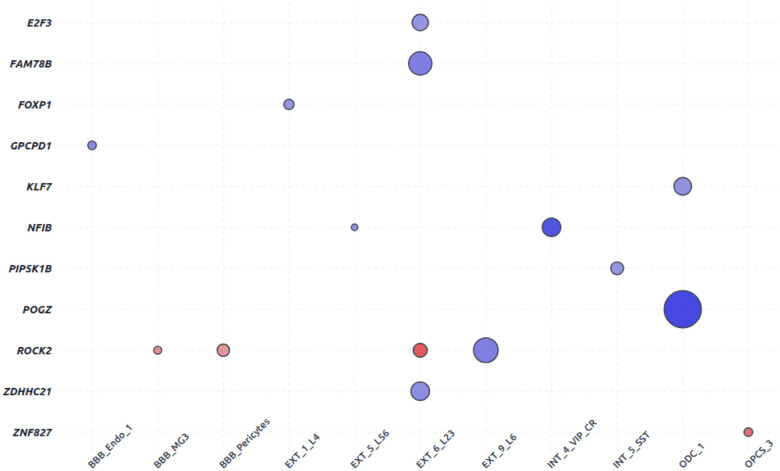
CLU UMAP projections of single-cell transcriptomes across four developmental timepoints (23 days, 1 month, 3 months, and 6 months postnatal). For each timepoint, the left panel shows the distribution of cells colored by gene expression density (purple blue = high, yellow = low), while the right panel displays cells colored by assigned cell type identity. Each point represents an individual cell, with spatial proximity indicating transcriptional similarity. The progressive expansion and refinement of cell type clusters across developmental stages demonstrates dynamic cellular maturation and increasing cellular heterogeneity. N = cells per timepoint. scRNAseq, single-cell RNA sequencing; UMAP, Uniform Manifold Approximation and Projection.

At the earliest timepoint (23 days), the cellular landscape displayed a relatively nascent organizational structure with several prominent cell populations. *CLU* transcriptional profile was dominated by apical radial glia (aRG) and cortical hem cells, which exhibited the most prominent expression levels across the tissue. These populations represented the primary progenitor pools driving early neurogenesis and regional patterning of the developing brain. By 1 month postnatal, substantial reorganization of the cellular architecture was apparent. The tissue showed increased representation of maturing glial populations, with distinct clusters of astrocytes and expanding oligodendrocyte lineage cells at various differentiation stages. The persistence of *CLU* expressed radial glia (RGL) and substantial progenitor populations indicated their role in the emergence of maturing glial cell populations. At 3 months, astrocytes had become one of the dominant cell types, robustly expressing *CLU* and showing clear transcriptional maturation compared to earlier developmental stages. By 6 months, the cellular architecture had achieved a mature, stable configuration characteristic of the adult brain. The UMAP projection showed well-defined, compact clusters with minimal overlap, indicating distinct transcriptional identities. Apical radial glia and mature astroglia remained the major populations expressing *CLU*, highlighting the continued importance of this gene in maintaining glial homeostasis and function in the mature tissue (Fig 8).

### Convergence of regulatory and coding variants in known neurodevelopmental genes

Remarkably, our analysis revealed that the most relevant genes affected by *de novo* regulatory variants corresponded to genes previously identified as harboring pathogenic variants in exome sequencing studies. Among the identified genes with a remarkable trajectory in single-cell expression, were *ROCK2* and *NFIB,* both with previouslyestablished roles in brain development and have been previously associated with NDDs through loss-of-function or pathogenic missense variants^16^.

This finding suggests that these genes may exhibit marked dosage sensitivity, where both alterations in protein sequence (exonic variants) and perturbations in expression levels (regulatory variants) result in similar phenotypes. scRNA-seq expression analysis revealed that these genes display highly specific spatiotemporal expression patterns during brain development, suggesting that precise regulation of their expression is essential for proper brain development and function.

## DISCUSSION

We have analyzed targeted sequencing data from 200 ASD trios, where *de novo* mutations and large CNVs, crucial in regulation of gene expression, had eluded detection in prior exome sequencing and microarray analyses^14^. Moreover, we have applied the Sei framework, which is the most comprehensive chromatin-level sequence model to date. Importantly, in terms of general conclusions: we have identified that 28% of *de novo* variants and 25% of inherited variants exhibit a high regulatory potential in patients with negative results from WES and microarray analysis, as assessed by Sei. The primary limitation of the current genetic cohort is the absence of a sibling or neurotypical control group. Future work should focus on incorporating additional data from an appropriate control cohort to overcome this constraint. Ideally, access to larger ASD cohorts would also increase statistical power, enabling the extension of the current findings from inherited variants to de novo variants (DNVs), which are currently limited in number. At the same time, it is important to note that this cohort consists of individuals diagnosed with ASD who were preselected based on negative exome sequencing and CNV array results. This stringent preselection enhances the likelihood of identifying non-coding regulatory contributions but also represents a unique feature that is not replicable in most publicly available genetic cohorts.

### Exploring the sex transmission bias in regulatory variation

In our cohort, the male-to-female ratio was 4.0, consistent with previous findings^28^. This ratio persisted even when prioritizing high-impact regulatory variants, with a ratio of 3.7 within *de novo* variants and 4.03 within inherited variants. However, in evaluating transmission bias for ultra-rare inherited variants, we detected a statistically significant transmission bias favoring inheritance from the mother that remained significant even when the analysis was limited to autosomes.

The debate surrounding the excess of germline DNMs arising on the maternal chromosome and the transmission bias favoring maternal inheritance has been a longstanding and essential aspect of ASD etiology^4,31,37,38^. Theoretically, if females exhibit reduced vulnerability to ASD, as evidenced by the consistently observed female-to-male ratio of 4:1 in this and most ASD studies, and individuals with ASD experience reduced fecundity, basic genetic principles predict that mothers are more likely sources of risk alleles than fathers. Initial observations relating to the observed sex bias indicated a trend of excess maternal *de novo* CNVs, although the pattern did not achieve statistical significance. Notably, females with ASD exhibited a higher number of *de novo* CNVs compared to males, and these genomic imbalances were both larger and impacted a greater number of genes^39^. Later, some of the first statistically significant genetic evidence of a transmission bias from mothers to their sons was observed for protein-coding SNVs in the study conducted by Krumm *et al*.^40^. On the contrary, one study identified a significant paternal-origin effect for CRE-SVs^4^. One possible explanation offered for the observation that mutations in regulatory regions follow an opposite pattern to what is predominantly seen in coding mutations, is that paternally inherited regulatory mutations, due to their less damaging impact, may require a greater number to manifest in disease. Crucially, this has given rise to an alternative hypothesis, suggesting that regulatory mutations may adhere to distinct principles compared to coding mutations.

Nevertheless, in line with our results, instances of the transmission of non-coding putative regulatory DNA from the mother to the male proband have been noted in genes such as *DSCAM* and *TRIO*, and both of them have been observed to be disrupted in ASD^1^.

Taken together, our results suggest that parent-of-origin effects may follow similar rules as coding variation. However, these results should be cautiously interpreted and the comprehensive contribution of a gender bias in non-coding variation to ASD needs to be determined through much larger studies. Still, this underscores that influences of sex bias on genetic risk for ASD are more intricate than previously understood, and the allelic spectrum of variants varies between the maternal and paternal genomes.

#### Sequence class dysregulation

Probably, the most important observation in this study is the implication of a global dysregulation of CTCF.

CTCF is reported as a high confidence gene (score = 1S) in SFARI, associated with Tourette syndrome^41^, ID^42^, and, more recently, with ASD^31,37,43^. In fact, it was identified by Zhou *et al*.^44^ as a gene reaching exome-wide significance in their analysis of rare *de novo* and inherited coding variants in 42,607 ASD cases, (p < 2.5 × 10^−6^) and thus included in our list of high risk ASD genes (Supplementary Table 3).

Interestingly, in our random selection of cCREs, only 2.6% were classified as CTCF in ENCODE (Figure 2 B), and this proportion drastically decreased when we filtered for *de novo* and inherited mutations. Moreover, we observed a significant depletion of CTCF cCREs in the final list of inherited variants (Table 2).

However, when prioritizing variants with a high regulatory impact, not only did their proportion increase, but they became the regulatory class most represented amongst the detected mutations. Specifically, an increased number of mutations was associated with a reduction (and not an increment) in the CTCF regulatory score, and this difference attained statistical significance for inherited variants. Although *de novo* variants, constrained by a limited statistical power in terms of numbers, did not reach significance, they showed a similar trend.

With this regard, it is important to note that the targeted sequencing approach followed in this chapter only covers 0.70% of the genome. Moreover, the selection of regulatory elements was performed in an arbitrary fashion, as it did not involve a priori hypotheses about which elements were most likely to influence gene expression. We solely restricted the regulatory elements to those active in tissues relevant to ASD. Thus, if we were to follow a WGS strategy, the results presented here suggest that we would likely obtain the same outcomes, supported with greater statistical power.

As further evidence, if we consider the top 10% of mutations with the highest class scores, they exclusively belong to the CTCF class, both for inherited and for *de novo* variants. Moreover, CTCF was one of the most frequently disrupted TF motifs in ASD cases *versus* controls in a single-cell analysis of gene expression and chromatin accessibility^34^, and in this study. This suggested a potential mechanistic impact on the chromatin architecture: deletion of CTCF binding sites or lost on CTCF binding affinity can lead to formation of new TADs, which can cause genes to contact enhancers normally spatially too far away to do so (a process known as “enhancer hijacking”)^34^.

Of note, Nakamura *et al*.^45^, published an analysis of promoter DNV from WGS data in a cohort of 5,044 ASD patients and 4,095 siblings. Interestingly, among the findings in their analyses, they specifically identified the enrichment of ASD-gene TAD promoters at CTCF-bound regions, further corroborating our results.

In addition, Nakamura *et al*.^45^, found a specific association of DNV in TADs containing ASD risk genes and identified specific TADs with enrichment of promoter DNVs. Thus, we aimed to assess whether the variants in each of the patients with elevated numbers of high impact inherited variants were located within the same and/or adjacent TADs. If this were to be corroborated, we could hypothesize that the loss of CTCF binding affinity in TADs relevant to ASD might collectively contribute to disease risk by simultaneously affecting the expression of pertinent genes. On the contrary, we found that these variants were present in different TADs. Still, these findings prove interesting as they offer mechanistic insights into how non-coding regulatory variation may be influencing ASD risk. Overall, it seems reasonable to assume that a substantial part of the non-coding genome in ASD is dysregulated in terms of its 3D-structure, and that further characterization of chromatin loop alterations (for example, by Hi-C analysis) will prove fruitful in further unraveling the missing heritability in ASD. Yet, it has been reported that CTCF contributes not only to the formation of TAD boundaries but also to the stabilization of promoter-enhancer interactions. Thus, it may also be possible that TAD boundaries remain unaffected, but changes in promoter targeting by enhancers (which is influenced by CTCF-binding) are responsible for the changes in gene expression leading to disease^46,47^.

### Candidate gene elucidation by physical distance and expression data

Our comparison of target gene assignments for cCREs carrying de novo and inherited mutations highlights the added value of incorporating functional data beyond simple genomic proximity. For de novo mutations, 7 out of 39 genes (~17,9 %) differed between genes assigned based on physical distance and those identified using gene expression and ChIP-seq data via T-Gene. This indicates that relying solely on proximity may miss a subset of functionally relevant target genes, as expression-based approaches can capture distal regulatory interactions that are not reflected by linear genomic distance. In the case of inherited mutations, the overlap between distance-based and expression-based gene assignments was higher (64.8%). Nevertheless, a substantial proportion (35.2%) of genes differed, suggesting that even for prioritized inherited variants, gene expression assignments can reveal additional candidate genes not detected by genomic proximity alone. Importantly, the use of T-Gene allowed further prioritization of candidate genes based on statistical significance (q-values and p-values), resulting in 74 inherited and 39 de novo candidate genes. This emphasizes that integrating functional genomic data provides a more nuanced view of regulatory variant impacts and enables more precise identification of candidate genes for further in silico studies and functional validation.

### Gene differential gene expression analysis using snRNAseq from ASD brain vs controls

Single-nucleus RNA-seq analysis in ASD brain cases vs controls for target genes revealed striking differences in the cellular contexts affected by de novo *versus* inherited regulatory variants.

De novo-associated genes showed more focused dysregulation patterns, with *POGZ* downregulation specifically in oligodendrocytes representing the strongest effect in our dataset. *POGZ* encodes a chromatin regulator essential for neurodevelopment, and its disruption has been associated with White-Sutton syndrome, characterized by intellectual disability and autism. In addition, *POGZ* was proved to have a key role in the developing forebrain and finds that it localizes to euchromatic regions and gene regulatory elements. Using Pogz knockout mice, it was found that *POGZ* promotes chromatin accessibility and drives transcription of clustered synaptic genes^48^.

Thus, the de novo gene expression analysis revealed alterations in a limited set of genes, with *POGZ* showing the most pronounced effect specifically in oligodendrocytes, while *ROCK2* and *NFIB* displayed more constrained cell type-specific patterns. This restricted profile suggests that de novo mutations may target critical developmental pathways with high penetrance effects in select cellular contexts, consistent with their typically more severe phenotypic consequences.

In contrast, inherited variants showed more widespread effects across multiple cell types, exemplified by *SYNJ2* downregulation across five distinct populations. *SYNJ2* encodes synaptojanin 2, a phosphoinositide phosphatase involved in synaptic vesicle recycling and clathrin-mediated endocytosis^49^. The bidirectional regulation of *UBC*,central to protein degradation pathways.across five cell populations—downregulated in layer 6 excitatory neurons but upregulated in interneurons, oligodendrocytes, layer 2/3 excitatory neurons, and oligodendrocyte precursors—illustrates the complexity of regulatory network disruption in ASD^50^. The inherited gene set also reveals extensive involvement of blood-brain barrier components, with significant alterations in pericytes, endothelial cells, and smooth muscle cells through genes like *CLU, DLC1,* and *TACC1*. This vascular involvement, absent from the de novo profile, suggests that inherited mutations may contribute to ASD through broader neurovascular dysfunction mechanisms. Thus, recent evidence suggests that vascular–neural interactions play a critical role in neurogenesis and cortical organization, providing key mechanistic insights and highlighting potential avenues for further research into NDDs^51^.

Layer-specific excitatory neuronal alterations further demonstrate the complexity of inherited effects, with genes like *DLC1* and *AKAP5* showing distinct patterns across different cortical layers. The involvement of diverse interneuron populations, microglia, and both mature and precursor oligodendrocytes in the inherited profile indicates a more systemic cellular disruption compared to the focused oligodendrocyte-centric pattern observed in de novo mutations.This differential cellular architecture suggests that inherited ASD risk operates through cumulative effects across multiple cell types and regulatory networks, while de novo mutations may act through more direct, high-impact disruptions of specific developmental processes. The broader cellular diversity in inherited mutations may explain the more variable expressivity and complex inheritance patterns observed in familial ASD cases.

To further contextualize the cell type–specific alterations identified in the snRNA-seq analysis, we next examined the developmental trajectories of these same de novo and inherited candidate genes in human cortical organoids.

### Analysis of Gene Expression in Cortical Organoids Across Developmental Stages

This analysis allows us to refine the importance of previously implicated genes by examining how their expression changes across cortical organoid stages that mirror human brain development (23 days, 1 month, 3 months, and 6 months). Notably, the developmental trajectories of the de novo genes *NFIB, POGZ,* and *ROCK2* show particularly strong relevance, while *CLU* emerges as the most prominent gene among the inherited variant set. These genes switch on and off at specific developmental stages, whereas the remaining genes—both de novo and inherited—show much more stable expression across organoid development.

The temporal expression dynamics of de novo versus inherited ASD risk genes reveal fundamentally distinct regulatory architectures during human cortical organoid development. De novo risk genes demonstrate progressive developmental activation, exemplified by *NFIB*'s linear upregulation and the striking bimodal expression of *POGZ* and *ROCK2* in ORG II cells. This biphasic pattern—with peaks at 1 month and 6 months flanking a 3-month trough—suggests these genes function during discrete developmental windows corresponding to initial cell fate specification and subsequent functional maturation. Such temporal specificity implies that de novo mutations may disrupt development at precise neurodevelopmental checkpoints, potentially explaining their high penetrance and phenotypic severity.

In contrast, inherited ASD risk genes maintain constitutive expression across developmental timepoints, consistent with their roles in essential cellular housekeeping and synaptic function. The near-uniform expression of genes like *UBC* among others suggests these represent core neurodevelopmental machinery under strong purifying selection. *CLU*'s intermediate expression pattern—consistently lower than other inherited genes yet increasing at later stages—may reflect a specialized regulatory role balancing constitutive function with developmental modulation, possibly in protein homeostasis or synaptic pruning during circuit refinement.

These contrasting patterns support a model wherein de novo mutations affecting temporally regulated developmental programs produce severe, early-onset phenotypes through disruption of critical specification windows, while inherited variants in constitutively expressed genes likely require additional genetic or environmental factors to manifest clinical phenotypes, consistent with their lower penetrance and more variable presentation in ASD populations.

### Connecting CTCF Regulatory Variants to Cell-Type-Specific Expression Dysregulation

The predominant disruption of CTCF-binding sites observed in our regulatory variant analysis (53.19% of high-impact de novo variants and 40.62% of high-impact inherited variants) provides a framework for understanding the cell-type-specific and temporally dynamic expression changes detected in our downstream functional analyses. Integration with single-cell transcriptomics provides key insight into how architectural perturbations at CTCF sites translate into cellular phenotypes.

De novo regulatory variants—targeting expression of genes such as *POGZ*, *ROCK2*, and *NFIB*—resulted in focused dysregulation within oligodendrocytes and selected neuronal populations. These cell types exhibit strong reliance on precise temporal and spatial transcriptional control during cortical development. The enrichment of de novo variants at CTCF-boundaries likely alters enhancer adoption or boundary insulation specifically in these lineages, leading to lineage-selective transcriptional vulnerability. In contrast, inherited variants mapped to genes with broad constitutive expression (e.g., *SYNJ2*, *UBC*, *CLU*, *DLC1*), producing diffuse transcriptional alterations across neurons, glia, and blood–brain barrier components. This dual pattern suggests that the degree of cellular specificity emerging from CTCF perturbation reflects the underlying regulatory architecture and dosage sensitivity of the affected genes.

Cortical organoid models further revealed that de novo and inherited genes differentially impact the temporal dynamics of neurodevelopment. Genes affected by de novo variants displayed marked developmental modulation, with *POGZ* and *ROCK2* showing bimodal activation peaks in outer radial glia and *NFIB* exhibiting linear upregulation across progenitor populations. Such tightly orchestrated expression trajectories are known to be dependent on intact chromatin boundaries, providing a mechanistic link between CTCF disruption and temporally restricted cellular effects. In contrast, inherited variant–associated genes maintained constitutive expression across developmental stages, with *CLU* as the only gene demonstrating late-stage regulatory induction. Importantly, the identification of de novo regulatory variants affecting established ASD risk genes *POGZ*, *NFIB* extends the genetic architecture of ASD beyond coding mutations. These findings collectively indicate that CTCF-mediated regulatory disruption represents a unifying mechanism through which both de novo and inherited rare variants contribute to ASD, albeit with distinct developmental trajectories and cellular consequences.

In the case of *POGZ*, it is a well established autism risk that promotes chromatin accessibility and CTCF functions as a key recruiter of chromatin regulators. In this context, CTCF loss of function—such as through regulatory variants that reduce its DNA-binding affinity—could contribute to *POGZ* dysregulation. Together, these mechanisms may suggest a critical regulatory *CTCF–POGZ* axis^52,53^.

### Convergence of regulatory and coding variants in known neurodevelopmental genes

The convergence of de novo regulatory variants with established ASD risk genes provides compelling evidence for dosage sensitivity as a critical pathogenic mechanism in neurodevelopmental disorders. Our analysis revealed that genes affected by de novo regulatory variants substantially overlap with those harboring pathogenic coding mutations identified in large-scale exome sequencing studies. Notable examples include *POGZ*, and *NFIB*. This concordance suggests these genes operate within narrow expression windows where both protein-level alterations and transcriptional dysregulation converge on similar phenotypic outcomes.

*POGZ* was identified in the large exome-sequencing study by Satterstrom et al. (2020), where it emerged as one of the top ASD-associated genes^16^. Consistent with this strong genetic evidence, *POGZ* is currently classified in the SFARI Gene database with a high-confidence 1S score. In the case of *NFIB*, a de novo loss-of-function variant was first identified in an ASD proband from the Simons Simplex Collection^54^. Subsequently, 18 individuals with *NFIB* haploinsufficiency—10 carrying microdeletions involving *NFIB*, five with truncating variants, and three with missense variants that markedly reduced transcriptional activity were reported ^55^. These individuals presented with mild intellectual or learning disability, speech delay, and macrocephaly, and behavioral abnormalities were frequent; notably, five cases exhibited ASD or autistic features.

Single-cell expression profiling demonstrated that these dosage-sensitive genes exhibit precisely choreographed spatiotemporal dynamics, with *POGZ* and *ROCK2* showing bimodal expression peaks in ORG II cells during critical specification windows at 1 and 6 months, while *NFIB* displays progressive upregulation across multiple progenitor populations. Such exquisite temporal and cell-type specificity underscores why these genes are vulnerable to regulatory disruption—even modest expression perturbations during developmental windows may fundamentally alter cell fate decisions and circuit assembly.

These findings suggest a substantial fraction of genetically undiagnosed ASD cases may harbor regulatory variants affecting the same core pathways disrupted by coding mutations. This regulatory variant burden likely contributes to the “missing heritability” in ASD and highlights the critical need for genome-wide sequencing strategies coupled with functional interpretation frameworks capable of identifying and prioritizing non-coding variants.

Ultimately, this research supports a model in which ASD arises from disruption of multiple converging biological pathways, with regulatory variants contributing through subtle, cell-type-specific alterations in gene expression that accumulate to perturb critical neurodevelopmental processes. This nuanced understanding of genetic risk moves beyond simple single-gene models and emphasizes the importance of considering cellular context, developmental timing, and pathway-level effects when interpreting genetic variation in complex neurodevelopmental disorders.

## Supplementary Files

This is a list of supplementary files associated with this preprint. Click to download.

- SupplementaryTables.xlsx
- Supplementarymaterial.docx

Supplementary information The online version contains supplementary material available at https://

## Figures and Tables

**Table 1.** Enrichment of cCREs in *de novo* and inherited variants.

cCRE	Original selection	De novo	p-value	Inherited	p-value
dELS	31,023	52	0.25	19,027	0.09
pELS	30,799	58	0.92	16,510	<2.2 × 10^−16^
PLS	20,306	51	0.02	15,616	<2.2 × 10^−16^
CTCF	2,260	2	0.37	1,265	1.46 × 10^−3^
DNase-H3K4me3	1,006	1	0.75	605	0.55

**Table 2.** Enrichment of variant scores within each of the regulatory classes, divided in positive and negative effects.

Regulatory class	*De novo*		p-value	Inherited		p-value
	+	-		+	-	
CTCF CTCF Cohesin	10	15	0.42	2489	2896	**1.55 × 10** ^**−8**^
E4 Multi-tissue	5	3	0.73	1413	1930	**<2.2 × 10** ^**−16**^
E5 B cell like	2	1	1.00	445	599	**1.05 × 10** ^**−6**^
E12 Erythroblast like	2	1	1.00	430	588	**4.11 × 10** ^**−7**^
P Promoter	2	0	0.50	246	455	**1.24 × 10** ^**−15**^
TF3 FOXA1 / AR / ESR1	1	0	1.00	312	378	**6.64 × 10** ^**−3**^
E6 Weak epithelial	-	-	-	97	107	0.53
TF5 AR	1	1	1.00	85	69	0.23
L5 Low signal	-	-	-	74	54	0.09
E7 Monocyte / Macrophage	-	-	-	65	62	0.86
E11 T cell	-	-	-	44	39	0.66
E9 Liver / Intestine	0	1	1.00	15	44	**1.02 × 10** ^**−4**^
PC4 Polycomb / Bivalent stem cell Enhancer	-	-	-	29	36	0.46
E1 Stem cell	1	0	1.00	28	33	0.61
TF2 CEBPB	1	0	1.00	20	26	0.46
E2 Multi-tissue	-	-	-	19	23	0.64
TF4 OTX2	-	-	-	21	12	0.16
E10 Brain	-	-	-	4	10	0.18
E8 Weak multi-tissue	-	-	-	4	7	0.55
TN1 Transcription	-	-	-	5	4	1.00
TN3 Transcription	-	-	-	5	3	0.73
E3 Brain / Melanocyte	-	-	-	4	4	1.00
PC1 Polycomb / Heterochromatin	-	-	-	4	2	0.69
HET4 Heterochromatin	-	-	-	2	3	1.00
TN4 Transcription	-	-	-	2	3	1.00
PC2 Weak Polycomb	-	-	-	2	2	1.00
PC3 Polycomb	-	-	-	2	0	0.50
HET5 Centromere	-	-	-	0	1	1.00
HET1 Heterochromatin	-	-	-	1	0	1.00
TN2 Transcription	-	-	-	0	1	1.00

## Data Availability

All data generated as part of the ASC (Autism Sequencing Consortium) were transferred to dbGaP with Study Accession: phs000298.v4.p3. Targeted sequencing data (regulatory regions) from the Spanish cohort can be found at EGA, the European Genome-phenome Archive ( Title and number of the dataset: EGAS50000001395 Targeted sequencing data of regulatory regions in 200 Spanish ASD trios. https://ega-archive.org/studies/EGAS50000001395)
